# “Cerebellar contribution to visuo-attentional alpha rhythm: insights from weightlessness”

**DOI:** 10.1038/srep37824

**Published:** 2016-11-24

**Authors:** A. M. Cebolla, M. Petieau, B. Dan, L. Balazs, J. McIntyre, G. Cheron

**Affiliations:** 1 Laboratory of Neurophysiology and Movement Biomechanics, CP640, ULB Neuroscience Institute, Université Libre de Bruxelles, 1070 Brussels, Belgium; 2 Inkendaal Rehabilitation Hospital, 1602 Vlezenbeek, Belgium; 3 Institute for Psychology of the Hungarian Academy of Sciences, Department of Experimental Psychology, 1132 Budapest, Hungary; 4 LNRS/CNRS-Université René Descartes Paris V, 75006, Paris, France; 5 Laboratory of Electrophysiology, Université de Mons, 7000 Mons, Belgium

## Abstract

Human brain adaptation in weightlessness follows the necessity to reshape the dynamic integration of the neural information acquired in the new environment. This basic aspect was here studied by the electroencephalogram (EEG) dynamics where oscillatory modulations were measured during a visuo-attentional state preceding a visuo-motor docking task. Astronauts in microgravity conducted the experiment in free-floating aboard the International Space Station, before the space flight and afterwards. We observed stronger power decrease (~ERD: event related desynchronization) of the ~10 Hz oscillation from the occipital-parietal (alpha ERD) to the central areas (mu ERD). Inverse source modelling of the stronger alpha ERD revealed a shift from the posterior cingulate cortex (BA31, from the default mode network) on Earth to the precentral cortex (BA4, primary motor cortex) in weightlessness. We also observed significant contribution of the vestibular network (BA40, BA32, and BA39) and cerebellum (lobule V, VI). We suggest that due to the high demands for the continuous readjustment of an appropriate body posture in free-floating, this visuo-attentional state required more contribution from the motor cortex. The cerebellum and the vestibular network involvement in weightlessness might support the correction signals processing necessary for postural stabilization, and the increased demand to integrate incongruent vestibular information.

Brain oscillations underlie brain function and characterise brain states^1^. In both the cerebral and cerebellar cortex, oscillations are essential to ensure complex and integrative functions. Oscillations represent “universal codes” based on which communication is established in the central nervous system by local-global communication that takes into account pre-existent endogenous ongoing constraints^1^,^2^,^3^,^4^. Brain dynamics may be non-invasively approached, including in the cerebellum, thanks to electroencephalography (EEG)^5^,^6^. EEG oscillations represent direct signals from the global electrical neural activity. In a state of relaxed wakefulness when the eyes are closed, the power of the alpha (8–12 Hz) rhythm increases in the occipital and parietal scalp areas^7^. Alpha rhythm relates to the maintenance of the neural network coherence in the absence of visual and sensorimotor information and is considered as marker of cortical inactivity or index of the active inhibition of sensory information^8^. The power of the alpha rhythm decreases when opening the eyes, which is interpreted as reflecting entrance of visual (and sensorimotor) information^9^. Similarly, the states of increased attention and sensory perception are associated with a decrease in alpha power, which is thought to reflect the release of inhibitory control or “cortical excitation”^10^. More precisely anticipatory attention in the absence of stimulation is related to the diminution of alpha power reflecting the maintenance of the target information or the selective activation of the knowledge system^10^. The influence of weightlessness on the dynamic organization of the brain is a central issue for space neuroscience and has important implications for future long-term missions. It also helps clarify a number of issues in neuroscience^11^. Microgravity offers an environment where cognitive and sensorimotor functions are continuously challenged. The absence of graviception affects the vestibular information, which in turn disturbs the dynamic balance with the visual and the proprioception information that guarantees the coherent internalisation of gravity in the human brain processing^12^. In the same line, it has been recently shown that gravity-related sensory inputs exert a top-down modulation on visual inputs involved in virtual 3D navigation that is suppressed during microgravity^13^. In microgravity the multiple sensory inputs must be dynamically re-weighted in order to maintain the behavioural goals^13^. The central nervous system must properly adapt in order to organize the control of functions such as posture, eye-hand coordination, spatial orientation and navigation^14^. Astronauts’ performance remains good after a variable period of adaptation^12^,^15^,^16^,^17^. Thus microgravity offers an environment pushing the regulatory process exerted on the different inputs to the cortex at the basis of adaptation. Previous EEG studies conducted in microgravity have mainly focused on sleep characterization^18^,^19^ and wakefulness alpha frequency band^20^. In this line, it has been demonstrated that the power of the alpha rhythm produced by eye-opening/closure state transition is potentiated in microgravity compared to the situation on Earth. This effect was not due to noise, haemodynamic effects or arousal. It was interpreted as an indicator of less connection with the environment^20^. In the present study, we focus on an exploratory^21^ watchfulness observational state^22^ through a visual-attention task performed in microgravity aboard the International Space Station (ISS) during long-duration spaceflights. We hypothesize that microgravity environment increases the global neural activity devoted to this task and thus expresses a stronger spectral power diminution of the alpha rhythm.

## Results

### Alpha power decrease is reinforced during weightlessness

**Figure 1:**
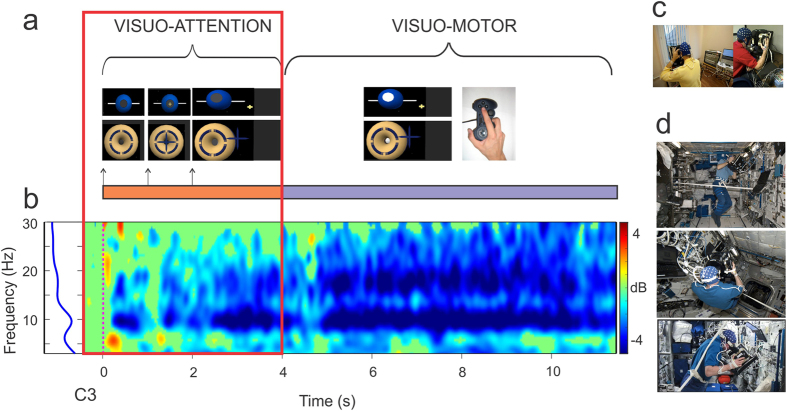
Experimental settings. (**a**) Scheme of task sequences. Note the two main “visuo-attention” and “visuo-motor” periods. (**b**) ERSP template for one representative subject during one on-ground (E_b_) session for EEG electrode C3. Note the blue mu ERD band during the “visuo-attention” reported in this study. (**c**) Recording set up on Earth and (**d**) during weightlessness. Note the free-floating.

**Figure 2:**
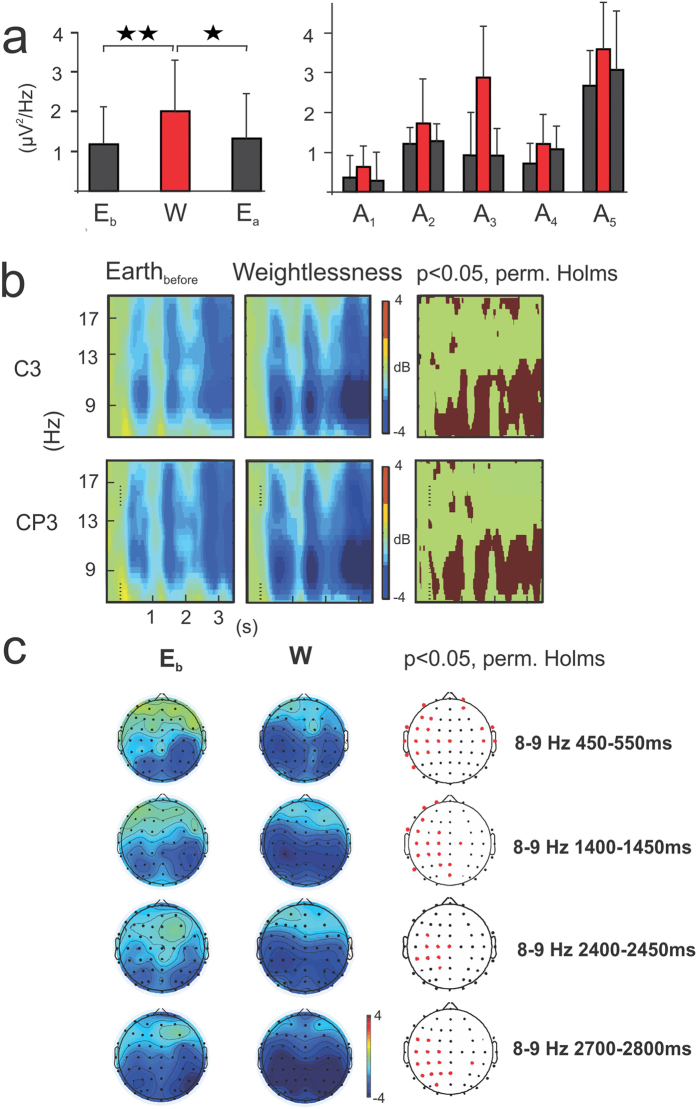
Mu ERD increases during weightlessness. (**a**) On the right, individual mu ERD (μV^2^/Hz) for each of the five astronauts for the three “E_b_”, “W” (in red bars) and “E_a_” conditions for EEG electrode C3 during the visuo-attentional period. On the left, global mu ERD for the “E_b_”, “W” (in red bars) and “E_a_” conditions for EEG electrode C3. Note that the mu ERD is highly significant and markedly increased with respect the two on-ground conditions (“E_b_” and “E_a_” respectively) (single-factor ANOVA test with 3 levels, Bonferroni corrected). (**b**) ERSP templates (n = 5) for C3 and CP3 EEG electrodes for “E_b_” and “W” conditions and their respective permutation Holms corrected statistics template. Note the darker blue mu band during weightlessness and its significant augmentation throughout the visuo-attentional period. (**c**) Topographical alpha-mu ERD distribution for “E_b_” and “W” conditions and their respective permutation Holms corrected statistics topographical template. Note that central contralateral (to subsequent movement) EEG electrodes showed significant differences between conditions all along the visuo-attentional period.

On Earth and in weightlessness, all astronauts showed a power decrease or event-related desynchronization (ERD) in the alpha frequency range with respect to the baseline prior to stimulus onset during the visuo-attentional task. This alpha ERD is indicated in the ERSP template by a blue band centered at 10 Hz (Fig. 1a). Additionally, a beta ERD band appears in the subsequent visuo-motor period. The individual alpha frequency ranges of interest at electrode C3 were: 8–12, 12–16, 8–12, 9–13, 7–11 Hz, for participants A_1,_ A_2,_ A_3,_ A_4_ and A_5_ respectively. All astronauts showed stronger alpha ERD in weightlessness (W) (2,01 ± 1.34 μV^2^/Hz ± s.e.m) with respect to that measured on Earth before (E_b_, 1.17 ± 0.99 μV^2^/Hz, p = 0.005,) and after (E_a_, 1.32 ± 1.11 μV^2^/Hz, p = 0.02) the flight (Fig. 2a, on the left) (single-factor ANOVA test with 3 levels, Bonferroni corrected, F (2, 222) = 5,4047) during the visuo-attentional period. The individual ERD estimations (μV^2^/Hz ± s.e.m.) were 0.31 ± 0.62, 1.21 ± 0.46, 0.93 ± 1.08, 0.72 ± 0.52, 2,67 ± 1,02 for E_b_, 0.64 ± 0.60, 1.73 ± 0.99, 2,88 ± 1.47, 1.21 ± 0.71, 3.59 ± 1.32 for W and 0.29 ± 0.75, 1.28 ± 0.48, 0.92  ± 0.80, 1.08 ± 0.72, 3.07 ± 1,63 for E_a_, for A_1,_ A_2,_ A_3,_ A_4_ and A_5_, respectively (Fig. 2a, on the right). The observed reinforcement of the alpha ERD for the W condition was not an effect of a one-side significant power spectrum increase in the baseline period. In fact, the absolute power spectrum during the baseline period of the W condition (1,82 ± 2.91 μV^2^/Hz) did not increase from E_b_ (2.85 ± 1.54 μV^2^/Hz), but presented a decreasing (not significant) trend (p = 0.11; F (2,222) = 4,9957). Along the same lines, the absolute power spectrum for the visuo-attentional period was significantly smaller for the W (−0.18 ± 1.80 μV^2^/Hz) than the E_b_ (1.7 ± 1.26 μV^2^/Hz) condition (p = 0.00000; F(2,222) = 20,489). The effect of the alpha ERD reinforcement in the W condition was similarly observed in the baseline-normalized spectrogram measurement, or ERSP (dB), calculated with permutation Holms corrected statistic (p < 0.05) at the C3 electrode (note the darker blue alpha band in the ERSP template of the W condition in Fig. 2b) and on the whole scalp (Fig. 2c). In weightlessness the alpha ERD reinforcement extended from the parietal and occipital areas to the central contralateral areas (mu ERD) throughout the visuo-attentional period (Fig. 2c).

### Motor cortex involved in the alpha power decrease during weightlessness

**Figure 3:**
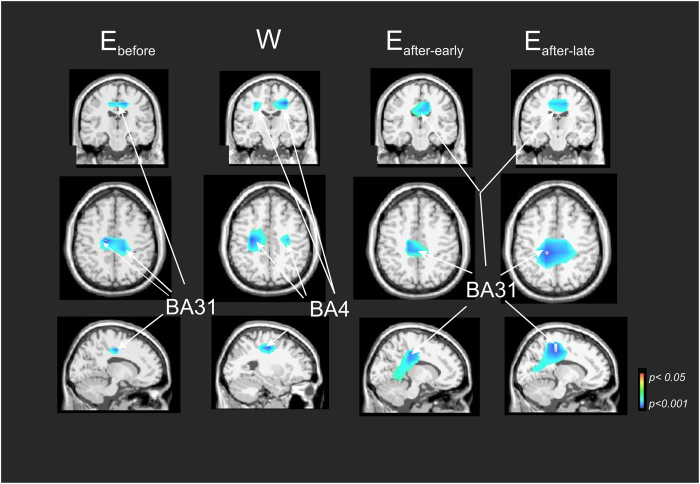
Alpha-mu ERD brain sources in E_b_, W, Earth_after-early_ and Earth_after-late_ conditions during the visuo-attention period. Nonparametric statistical maps calculated on the five astronauts for the alpha-mu ERD (with respect to the classical baseline as reference period). Each column represents the three views for a same spatial coordinate. Note bilateral motor cortex (BA4) activation during W condition.

In order to avoid the pitfalls occurring when focusing on the power spectrum topographies at the scalp level, associated with the mixing of multiple cortical processes by volume conduction^23^, we modelled the ERD brain sources by using the swLORETA method^24^,^25^. Figure 3 illustrates the nonparametric statistical source maps plotted for all participants at the ERD alpha frequency band during the visuo-attentional period, independently for E_b_, W, Earth_after-early_ and Earth_after-late_ conditions and with respect to the classical baseline as a reference period. On the ground before the flight (E_b_), the maxima of alpha ERD were localized in a single midline region of the posterior cingulate cortex (BA31, left cerebrum, −9.9, −21.3, 39.9 and BA31, right cerebrum, 14.1, −27.0 and 39.1). In contrast, in a condition of weightlessness (W), the maxima were lateralized in the motor cortex on both sides (BA4, left cerebrum, −25.0, −19.9, 40.0 and BA4, right cerebrum, 28.4, −18.0, 37.6). After the flight, the single midline region of the posterior cingulate cortex was obtained once again (BA31, left cerebrum, −1.6, −29.0, 34.6 for Earth_after-early_ condition) and (BA31, left cerebrum, −7.5, −35.4, 35 for Earth_after-late_ condition).

### Cerebellar contribution to the cerebral mu rhythm in weightlessness

**Figure 4:**
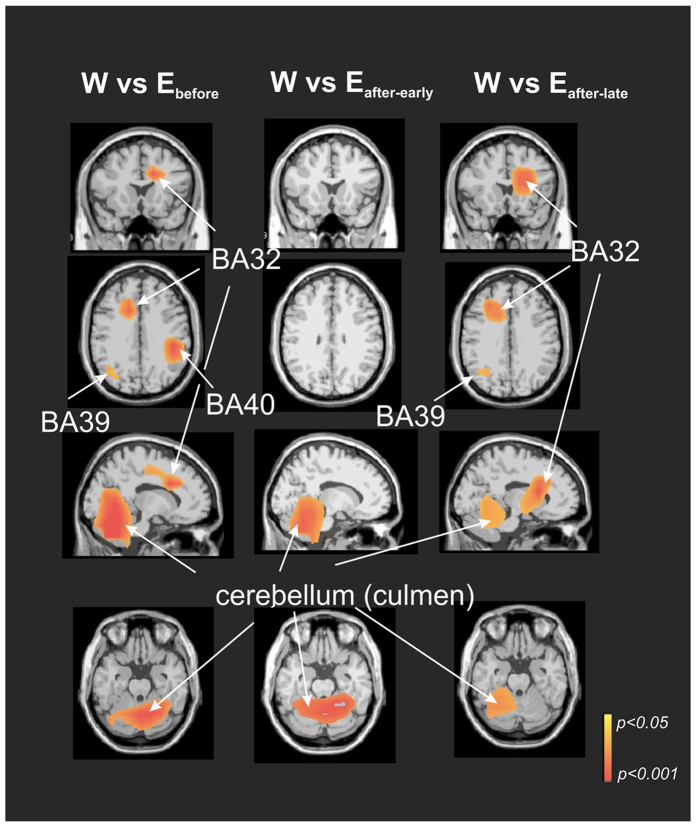
Alpha-mu ERD brain sources particular to weightlessness (“W”) condition. Nonparametric statistical maps of the alpha ERD sources characterising the weightlessness (“W”) contextual brain state. This was obtained by determining which sources were significantly more active in this condition with respect to each of the three others conditions performed on the ground (“W” > “Earth_before_”; “W” > “Earth_after-early_” and “W” > “Earth_after-late_”) during the visuo-attentional period. Note robust cerebellar activation in the three comparisons.

Figure 4 presents nonparametric statistical maps of the alpha ERD sources that characterize the brain state in the context of weightlessness (“W”). These were obtained by determining which sources were significantly more active in this condition with respect to each of the three other conditions observed/measured on the ground (“W” > “Earth_before_”; “W” > “Earth_after-early_” and “W” > “Earth_after-late_”) during the visuo-attentional period. The maxima of the alpha ERD in the “W” > “Earth_before_” condition were localized in the anterior cingulate gyrus (BA 32, left cerebrum, −17.3, 10.2, 30.9), middle temporal gyrus (BA 39, left cerebrum, −33.6, −68.4, 21.4), inferior parietal lobe (right cerebrum, BA40, 46.6, −32.4, 25.8) and cerebellum (right lobule V-VI, 5.9, −57.0, −12.2). The maximum of the alpha ERD in the “W” > “Earth_after-early_” condition was found in the cerebellum (right lobule V, 17.3, −46.3, −20.9), without any other equivalent maximum in the cerebral cortex. The maxima of the alpha ERD in the “W” > “Earth_after-late_” condition were found in the cerebellum (left lobule VI, −32.4, −51.8, −22.6), anterior cingulate gyrus (BA32, left cerebrum, −15.3, 9.2, 31.1) and parietal lobe (BA39, left cerebrum, −36.9, −60.4, 21.3). It is important to note that alpha ERD was localized in the cerebellum during the weightlessness condition when compared to pre-flight and (early and late) post-flight conditions.

## Discussion

In spite of the importance of graviception on Earth for visuo-motor and other aspects of motor control, the astronauts’ performance remained relatively good in microgravity (refs 11,12,15, 16, 17 for a review). Non-specific factors, such as the noisy environment in the ISS, stress, muscle artifacts and basic physiological factors (differences in blood circulation in both the brain and the body as a whole), seem unlikely to be the source of the alpha ERD reinforcement here described, as we did not find any significant variation when exclusively analyzing the power spectrum changes during the baseline period^13^,^20^.

During the Earth condition, at scalp level, the visuo-attentional period was characterized by a parietal-occipital alpha ERD. In our paradigm, the visuo-attentional period corresponds to a low level of attention process, as the participants were requested merely to observe the displayed scenario. This corresponds to an “exploratory state”^21^ or “watchfulness state”^22^. It was only after some seconds that the participants were required to interact by manipulating a joystick in a following visuo-motor docking. Thus the observed alpha ERD was not related to the subsequent finger (pre-) movement, as this began at least two seconds later^26^. Source analysis showed that the alpha ERD was localized in the posterior cingulate cortex (BA 31) consistently during all the “on Earth” conditions, i.e. before, early-after and late-after their stay in weightlessness. Interestingly, the posterior cingulate cortex is known to be the major node^27^ in the default mode network (DMN)^28^, whose activity corresponds to a state in which an individual is awake and alert, but not actively involved in a task that requires a high level of or is goal-directed^28^. This was the case during the visuo-attentional period. In addition, it has been hypothesized that the DMN supports a broad, low-level focus of attention when an individual monitors the external world in preparation for up- coming, self-relevant events before they happen^29^. This default state must be regarded as a complex situation involving the dynamic interplay between conscious and unconscious processes. It can be considered as a transient equilibrium, integrating all aspects of past history for use in the future for prediction^1^. DMN has been related to very slow (0.02–0.2 Hz) EEG band frequencies but also to the traditional EEG frequency bands (in the 0.5–45 Hz range)^30^,^31^. Alpha oscillations have been proposed as a correlate of DMN-related processes from a functional point of view^32^. States of increased attention (“exploratory state” included) and sensory perception are associated with a decrease in alpha power, which reflects the release of inhibitory control^10^. Further, alpha rhythms have been related to the posterior cingulate cortex during DMN activity^33^ and, together with low beta, these rhythms are reduced in posterior regions during the “resting state with eyes open” condition^34^.

During weightlessness, the alpha ERD significantly increased throughout the visuo- (low) attentional period, mostly at the central contralateral areas at scalp level, corresponding to the mu rhythm. Source analysis revealed that alpha-mu ERD during weightlessness was bilaterally localized in the primary motor areas. Thus in weightlessness, the activity of the DMN network that characterizes Earth condition, was outranked by the activity of the motor cortex. Interestingly astronauts were asked to not execute any explicit movement at this moment. As this is the initial period of the experimental trial after having accomplished the preceding one, they rather submerged themselves in the constraint of the experiment. We speculate that the activity measured in the bilateral motor cortex might be explained by the need to readjust or maintain an appropriate body posture when starting each experimental trial. Indeed, while gravity facilitates the automaticity of postural control on the ground, astronauts in the free-floating conditions aboard the ISS must continuously control their posture. The motor cortex is involved in postural control^35^ and its corticospinal tract neurons respond to postural changes even when muscle activity remains similar^36^. Postural control depends on the integration of afferent signals from the vestibular organs with visual, proprioceptive and tactile inputs^37^. Taking into account the fact that vestibular information is reduced because of microgravity and visual information is restricted because of the experimental task, finding alpha power suppression in the bilateral motor cortex may indicate a high responsiveness to somatic sensation^38^. Source analysis of the alpha range that characterizes the “weightlessness” condition revealed significant cerebellar activation with respect to the “Earth” condition. Cerebellar involvement, documented by source reconstruction, has been reported in other electroencephalogram investigations^5^,^6^,^39^. The fact that this cerebellar activation was not identified in the source analysis of the alpha-mu ERD with respect to the baseline (i.e. period preceding the visual stimulus onset) suggests that it was present during the whole testing period and it is not specifically related to the visuo-attentional task. Alpha and beta EEG rhythms have been identified in the cerebellum, the thalamus and a cortical network including the insula, cingulate and prefrontal cortex and show a correlation with fMRI signal fluctuations in the tonic alertness state^40^. Alpha oscillations in the cerebral cortex and alpha-beta oscillation in the cerebellar cortex follow similar behaviour in relation to movement preparation^41^. In both cortical circuits, the persistence of sensory or cognitive inputs is supported by neuronal membrane properties and by a recurrent chain of neurons that act as neural integrators^42^. Local circuits devoted to the ‘bottom-up’ processing of information coming from the environment are continuously controlled by the ‘top-down’ or ‘attentional’ influence exerted by the permanent state of activity^43^. In addition, the long range of bidirectional communications between the cerebral and cerebellar cortices assumes crucial functions in motor and cognitive behaviours^44^. The present finding of cerebellar activation may be explained by the specific requirements of the free-floating posture and motor program processes that involve postural stabilization and spatial orientation. It has been demonstrated in rodents, primates and humans that cerebellar oscillations may interact with cerebral oscillations^4^. Oscillations in cerebellar circuits could serve to coordinate their internal activity, but also to relate cerebellar activity to other distant cerebral areas^41^. In this way, specific theta/beta range oscillations (4–25 Hz) of the granule cell layer in the cerebellum have been linked to cerebral cortex activity^45^. These oscillations were first recorded in primates while immobile but attentive to the environment^46^. They lasted throughout the state of active or passive expectancy, linked to somatosensory and motor cortex rhythms and decreased at the beginning of a movement^41^. As “no movement” was explicitly required in weightlessness during this period, cerebellum alpha ERD may underlie the correction and error signals necessary to postural stabilization while free-floating. The (right) inferior parietal lobe (BA40), the (left) anterior cingulate gyrus (BA 32) and the (left) middle temporal gyrus (BA 39) were more active in weightlessness than on Earth. These areas together with both cerebellar hemispheres have been identified as part of the vestibular system in humans^47^ which is altered in weightlessness^14^. The alpha ERD recorded in this network may reflect a challenge of an increased demand to integrate partially reduced or incongruent vestibular information while free-floating^20^. From another point of view, an increased non spatial attentional demand in weightlessness corresponding to the task switching between the actual “visuo-attentional” and the subsequent “visuo-motor” periods or to the trial history due to the repetitive character of the task^48^ cannot be excluded as these regions also participate to the ventral attention system^49^.

## Conclusion

An alpha ERD potentiation was consistently observed in microgravity while executing a low-level visuo-attentional task. The bilateral motor cortex was involved in this effect, probably responding to the high demands of continuous readjustment or maintenance of an appropriate body posture while free-floating. In addition, the condition of microgravity was characterized by an alpha ERD in the cerebellum and other areas of the vestibular system, which might underlie the correction and error signals necessary for postural stabilization while free-floating as well as the increased demand to integrate partially reduced or incongruent vestibular information.

## Methods

### Participants

Five male astronauts (54.2 ± 2.6 years old) participated in this investigation. They were in excellent health, as regularly determined by a special medical commission throughout the experiment. Each astronaut was tested on the ground before flight, in weightlessness aboard the International Space Station (ISS) and on the ground after his return on Earth. The investigation was performed during the joint European-USA-Canadian Expeditions 20–21 and 34–35, and the joint European-USA Expeditions 26–27 and 30–31. All the missions involved a six-month long stay in orbit. The astronauts reported their use of medication and sleep quality.

The European Space Agency Medical Care Committee and the NASA Johnson Space Centre Institutional Review Board for Human Testing approved all experimental procedures, which were performed in accordance with the Helsinki Declaration of 1964. All participants gave written, informed consent prior to starting the experiment.

The detailed testing schedule was as follows. Prior to any EEG data recording, the astronauts got familiarized with the experimental tasks during two sessions of 60 min., separated by at least six days. The first EEG data collection took place at 66.8 ± 9.0 days before launch. This first session served as a general rehearsal and as one session’s worth of data to be used as back-up, if needed. Then, the astronauts were tested twice on Earth at 42.6 ± 0.9 and 28.0 ± 0.4 days before lift-of (“Earth_before_” or “E_b_”), twice during weightlessness aboard the ISS at 8.8 ± 1.8 and 54.6 ± 3.7 days of space flight (“Weightlessness” or “W”) and four times after their return (“Earth_after_” or “E_after_”), twice early at 3.0 ± 0.4 and 7.0 ± 1.2 days (“Earth_after-early_”) and twice later at 16.8 ± 0.64 and 20.2 ± 1.04 days following their landing (“Earth_after-late_”). The astronauts’ recordings on Earth_before_ and Earth_after_ were thus used as their own controls.

### Experimental set-up

Similar to previous space mission investigations^13^,^20^, the participants were told to look straight ahead at a laptop screen through a form-fitting facemask, fitted with a cylindrical viewing tube that removed external visual cues. The screen was centred on the line of sight, at a distance of ~30 cm from the eyes. The facemask was held firmly in place by a strap that passed behind the head. A joypad was mounted vertically on the right side of the cylindrical tube, allowing the participants to hold on to the entire structure (mask/tube/laptop) with both hands and still manipulate the joystick and press the buttons on the joypad with right thumb and right index finger.

On Earth, the participants performed the experiment while seated comfortably in front of the computer, which rested on a support table. The facemask was at eye level. During weightlessness in space flight, they performed the experiment in a free-floating condition. In order to restrain large shifts of trajectory, a belt was put around the subject’s waist and then attached to straps fixed by metal rings to the right and left racks of the Columbus module of the ISS.

### Stimuli and task

The participants observed a virtual environment displayed on the computer screen that simulated one of two scenarios: that of piloting a space ship towards the ISS or that of controlling a space ship from the ISS. In both randomly presented scenarios, their goal was to match positions between spaceship and ISS. There were 80 trials per session (40 for each scenario) and each trial lasted 15 seconds maximum. The 80 trials were divided in four blocks, which allowed the astronaut can take a break before starting the next block of 20 trials. At the beginning of each trial, the image of the space ship (or ISS) was presented first and the image of the ISS (or spaceship) one second after. One second after, the target deviated from the nominal straight-ahead position for two seconds. Throughout this first period (total duration of 4 seconds), the subject was asked to observe the space ship (or ISS) without performing any movement. In the present study, we report the EEG responses during this first “visuo-attentional” period. Six seconds after the presentation of the first image, the centre of the space ship (or ISS) subtly, but quite perceptibly, changed from white to grey, which indicated that the subject was required to take control of the spaceship and perform as quickly as possible (seven seconds maximum), and make the best possible manual adjustment to the space’s ship trajectory toward the target by pressing on a small joystick operated with the right index finger. Once the subject considered that he had matched the positions of both craft as closely as possible, he pressed a button on the joypad with his thumb. The centre of the space ship changed from white to blue or to yellow, depending whether the positions were matched acceptably (precision 3 mm) or not. The next trial started two seconds later. The entire second part constituted the “visuo-motor” period.

### EEG recordings

The EEG was recorded at a sampling rate of 1116 Hz (0.01–558 Hz band width) using the multi-electrode electroencephalogram mapping module (M.E.E.M.M) from the European physiology module installed in the Columbus module of the ISS, at the European Astronaut Centre (Köln, Germany) or in Star City (Moscow). In addition to the 58 EEG electrodes (from EEG cap with 10–20 electrode system placement), three electrooculogram (for horizontal and vertical EOG), one electrocardiogram and one electromyogram (from the first interosseous muscle of the right hand) signals were recorded. For some of the post-flight recordings at the Johnson Space Centre (Houston), the ANT system (The Netherlands) was used with a sampling frequency of 2048 Hz and with a resolution of 22 bits (71.5 nV per bit). An active-shield cap using 64 Ag/AgCl sintered ring electrodes and shielded co-axial cables (10–20 electrode system placements) was comfortably adjusted to the subject’s head.

All the electrodes were referred to the right earlobe. Scalp electrode impedances were measured and kept below 5 KΩ. Off-line, data treatment was performed using EEGlab^50^, ASA software (ANT system, The Netherlands) and in-house MATLAB-based tools.

The data were firstly resampled to a 512 Hz and the DC offset was removed. We analysed the single EEG trials (80 trials/session x 5 participants x 2 sessions per condition). Ocular artefacts were detected and removed by using the PCA method in ASA software where 95% of the calculated component explained the noise subspace. Any other remaining artefacts were rejected by careful visual inspection (no more than 8% of the total trials were eliminated). Each EEG trial contained samples from −1s before to 4 s after the first stimulus (space ship image) onset (“visuo-attentional” period).

### Event-related spectral perturbation

We used the EEGlab software for the time-frequency analysis of EEG oscillations. We calculated the baseline-normalized spectrogram or event-related spectral perturbation^51^ (ERSP in dB) for each of the 58 EEG signals, for each subject. ERSP measures variations in the power spectrum at specific frequency ranges of ongoing rhythms. These variations are related to the specific aspect of information processing that is time-locked to stimulus. We used wavelet transform for complex spectro-temporal representation, with Hanning-windowed sinusoidal wavelets at 3 cycles (lowest) to 25 cycles (highest). ERSP templates were calculated with 500 time points, using a window size of 571 samples (1115.23 ms) wide and estimating 100 frequencies from 3–50 Hz. For the significance level of ERSP bootstrap, resampling (p < 0.05) was used as a surrogate method. The significance between experimental conditions was calculated by permutation analysis (p > 0.05), with the Holms method for the correction of multiple comparisons^52^ (Fig. 2b,c).

We also calculated the absolute power spectrum values (FFT length of 512 data points) specifically for the frequency band of interest in each individual subject (5 bins in the alpha range) in the C3 EEG representative channel, separately for the baseline period (−1s to 0 s) and for the period after the first stimulus (space ship image) onset (0 s to 4 s), which we named the visuo-attentional period. We then calculated the subtraction of the obtained values in the baseline and visuo-attentional periods, yielding in the ERD estimations (μV^2^/Hz) ± s.e.m.). We made a statistical comparison of the measurements performed before the flight (“Earth_before_”), in flight (W) and after the flight (“Earth_after_”) with a single-factor ANOVA test with 3 levels and Bonferroni for correction of multiple comparisons (Fig. 2).

### Time-frequency source analysis

This method has been described in detail before^24^,^25^. We focused on the brain areas that exhibit ERSP around the frequency band of interest (specific for each subject). The analytic signal at the target centre frequency was first calculated for each EEG sensor channel for the nth trial of the experiment. We applied swLORETA^53^ (Standardized Weighted Low Resolution Electromagnetic Tomography which is based on sLORETA^54^) to the analytic signals for each individual trial; swLORETA has enabled the accurate reconstruction of surface and deep current sources in simulated data even in the presence of noise and when two dipoles are simultaneously actives, by incorporating a singular value, decomposition-based lead field weighting that compensates for the varying sensitivity of the sensors to current sources at different depths. The ERPS in brain space over the n trials were then calculated, as proposed by Lin *et al.*^55^. The solution was computed using 2030 voxels (5.00-mm grid spacing) and was restricted to the grey matter of the cerebrum and cerebellum based on the probabilistic brain tissue maps available from the MNI. The solution was computed during the 4 s after the first stimulus onset, referred to as the “active period” or “visuo-attentional period”, and during the baseline period (−1s before the “stimulus onset), referred to as the “reference period”. Finally, voxels and the recording array (electrodes) were placed in registration with the Collins 27 MRI produced by the Montreal Neurological Institute^56^. The Boundary Element Model (BEM) was used for solving the forward problem. Talairach coordinates were obtained for every voxel by placing the corresponding Talairach markers onto the Collins anatomical template^57^. The final coordinates of the maxima values (x,y,z, Talairach coordinates) we provided for labelling the corresponding brain areas were based on the Talairach atlas.

For our statistical analysis, we used the nonparametric permutation method^58^ to identify the ERSP sources^24^. As a first step, we used the classical baseline period preceding the stimulation as a reference (1 s before stimulus onset) and the “visuo-attentional” period as the active condition (4 s after the stimulus onset) in order to calculate nonparametric statistic maps, which represent specific brain sources of the ERSP in each condition (“Earth_before_”, “W”, “Earth_after-early_” and “Earth_after-late_”) for the whole population. In a second step, we computed nonparametric statistic maps comparing the “visual-attentional” periods of each condition. This allowed us to specify which sources were more active in the condition of weightlessness (“W” > “Earth_before_”; “W” > “Earth_after-early_”; “W” > “Earth_after-late_”) for characterizing this contextual “brain state” (weightlessness versus on the ground).

We used paired t-test for swLORETA solutions to compare the active versus reference conditions in each subject, with a null hypothesis corresponding to the absence of difference between the active and reference conditions, which is equivalent to stating that the distribution of the voxel values of the participants’ difference inverse solution images has a zero mean. We used the 95th percentile of the permutation distribution for the maximal statistics which defines the 0.05 level of the corrected significance threshold. In other words, we can reject the null hypothesis for any voxel with t-values of the un-permuted T image greater than the 95th percentile of the permutation distribution of the maximal statistics^58^.

## Additional Information

**How to cite this article**: Cebolla, A. M. *et al.* “Cerebellar contribution to visuo-attentional alpha rhythm: insights from weightlessness.” *Sci. Rep.* **6**, 37824; doi: 10.1038/srep37824 (2016).
